# An improved method to study *Phytophthora cinnamomi* Rands zoospores interactions with host

**DOI:** 10.1186/s12870-024-05205-2

**Published:** 2024-06-06

**Authors:** Lucía Del Castillo-González, Serine Soudani, Noelia De La Cruz-Gómez, José Antonio Manzanera, Marta Berrocal-Lobo

**Affiliations:** Centro para la Biodiversidad y Desarrollo Sostenible (CBDS), ETSIMontes, Forestal y del Medio Natural, Universidad Politécnica de Madrid, Ciudad Universitaria s/n, Madrid, 28040 Spain

**Keywords:** *Phytophthora cinnamomi* Rands, Zoospores, *Solanum lycopersicum*, qRT-PCR, *Quercus Sp*

## Abstract

*Phytophthora cinnamomi* Rands is a highly prevalent phytopathogen worldwide, ranking among the top ten in terms of distribution. It inflicts crown rot, canker, and root rot on numerous plant species, significantly impacting the biodiversity of both flora and fauna within affected environments. With a host range spanning over 5,000 species, including important plants like *Quercus suber, Quercus ilex, Castanea sativa*, and commercially significant crops such as avocado (*Persea americana*), maize (*Zea mays*), and tomato (*Solanum lycopersicum*), *Phytophthora cinnamomi* poses a substantial threat to agriculture and ecosystems. The efficient dissemination of the oomycete relies on its short-lived asexually motile zoospores, which depend on water currents to infect host roots. However, managing these zoospores in the laboratory has long been challenging due to the complexity of the life cycle. Current protocols involve intricate procedures, including alternating cycles of growth, drought, and flooding. Unfortunately, these artificial conditions often result in a rapid decline in virulence, necessitating additional steps to maintain infectivity during cultivation. In our research, we sought to address this challenge by investigating zoospore survival under various conditions. Our goal was to develop a stable stock of zoospores that is both easily deployable and highly infective. Through direct freezing in liquid nitrogen, we have successfully preserved their virulence. This breakthrough eliminates the need for repeated culture transfers, simplifying the process of plant inoculation. Moreover, it enables more comprehensive studies of *Phytophthora cinnamomi* and its interactions with host plants.

**Supplementary Information:**

The online version contains supplementary material available at 10.1186/s12870-024-05205-2.

## Introduction

The oomycete *Phytophthora cinnamomi* Rands stands out as one of the most destructive phytopathogens known, with a vast host range exceeding 5000 species across herbaceous, forest, and non-forest ecosystems. Its infections lead to severe diseases and extensive ecological and economic losses [1–4]. Compounded by factors linked to global warming, such as environmental shifts, *P. cinnamomi* incidence is on the rise [1, 5–10]. Extreme drying and warming temperatures are unfavorable conditions for Phytophthora species and other related subspecies [1, 9, 11–14]. To confront this growing threat, new research methodologies are imperative. These methods must enable comprehensive studies of *P. cinnamomi* to understand its behavior and combat its spread effectively in the years to come.

*Phytophthora cinnamomi* was initially identified as the causative agent of stripe canker in cinnamon trees (*Cinnamomum burmanii*) in Sumatra, leading to its classification as an oomycete [15]. It has since been linked to sporadic mortality among species of the Proteaceae species [16, 17]. Additionally, *P. cinnamomi* has been associated with infections caused by *Phytophthora cambivora* in chestnut trees and various other hosts since as early as 1966 [18], and even earlier in American chestnut (Castanea dentata) before 1910 [19, 20]. However, recent research suggests that the species’ origin may not be in Sumatra [21] but possibly in Papua New Guinea [22]. Its spread has extended to other regions in eastern and southern Asia, as well as New Guinea and Australia [22, 23].

*Phytophthora cinnamomi* exerts a significant negative economic impact, leading to the widespread mortality of native plants globally. Its effects extend beyond mere economic concerns, contributing to alterations in vegetation communities and a decline in botanical diversity. This reduction in plant diversity has ripple effects, affecting food availability for various wildlife species, ultimately impacting populations of birds and small mammals. Thus, the repercussions of *P. cinnamomi’s* presence are far-reaching, affecting both ecosystems and economies worldwide [24]. . In the USA, *P. cinnamomi* was first identified in a commercial blueberry plantation in Arkansas in 1978 [25]. Since then, it has been extensively documented on American white oak (*Quercus alba*) in the Appalachian Mountains and has inflicted substantial damage to avocado trees in California [26]. In southern regions of Europe, including Portugal, Spain, France, and Italy, *P. cinnamomi* targets native chestnut trees (*Castanea sativa*), mature cork oaks (*Quercus suber*), and holm oaks (*Quercus ilex*), among other species [20, 27–29]. Its presence in these areas sometimes leads to significant market impact due to the damage caused [30]. For instance, the production of acorns from holm oaks in meadows serves as the primary feed for Spanish acorn-fed ham, underscoring the potential impact of *P. cinnamomi* on this market [27, 31–33]. . *P. cinnamomi* is responsible for inducing severe forest dieback, known as Jarrah dieback, in the northern jarrah forest of Western Australia [34]. This destructive phenomenon significantly impacts macrofungal diversity in the region [35, 36]. With the onset of global warming and rising temperatures, *P. cinnamomi* is expanding its reach into central, colder regions of Europe, such as Germany and Poland. Recent detections of *P. cinnamomi* in the commercial sector, particularly in *Vaccinium corymbosum* (blueberry) nursery plants, underscore the growing threat posed by this pathogen [9]. In south-western Australia, a widespread forest die-off in 2011 coincided with exceptionally hot and dry conditions, occurring within an ecosystem historically affected by *P. cinnamomi* root disease, also known as Phytophthora dieback [34, 37]. Given the current scenario, combatting *P. cinnamomi* has become an urgent priority, necessitating concerted efforts to mitigate its effects and protect ecosystems [1, 2, 4, 38].

The most virulent form of *P. cinnamomi* is its asexual biflagellate zoospores, which possess a remarkable capacity for dispersal and survival in water [1, 4, 39–41]. During spring and high humidity seasons, following periods of drought, heavy rainfall can lead to soil waterlogging, creating favorable conditions for zoospores. Under these circumstances, zoospores propel themselves using flagella, swimming rapidly (up 250 mm/s) in both forward and backward directions, with a strong attraction to root chemicals. They bind to roots via annexin proteins [39, 42–44], and a well-known mechanism involving biofilm formation [39, 41, 45, 46]. Once attached to the roots, colonization proceeds, leading to root necrosis and ultimately impairing water supply to the aerial parts of the plant [4, 46].

Despite the wealth of research articles addressing *P. cinnamomi* [4, 47], and its increasing incidence [1, 30, 48, 49], laboratory protocols for handling this complex oomycete and its zoospores have seen minimal evolution since 1965 [6, 24, 50–55]. There exists significant variability in laboratory conditions for obtaining zoospores from *P. cinnamomi* mycelium and in plant inoculation methodologies. The primary methods involve inoculating fragmented plant tissues with mycelial fragments or culture filtrates, using cut leaves, roots, shoots, or explants [56–58]. Assays on cut plants hold commercial significance due to the high prevalence of *P. cinnamomi* in flowering ornamental plants [56, 59–61]. Alternatively, assays utilizing freshly obtained zoospores involve inoculating plant seedlings or plantlets, sometimes obtained through lengthy embryogenesis processes requiring months of micropropagation of forest species [28, 62–66]. In both cases, researchers face considerable time investment not only in acquiring explants but also in obtaining fresh zoospores.

In this study, we have developed a straightforward methodology for obtaining and conserving stocks of zoospores with a high infectivity capacity, thereby enabling researchers to conduct less time-intensive assays while preserving phytopathogen virulence. Our findings have introduced two additional steps to existing protocols, facilitating the use of zoospore stocks for repetitive assays. We tested the infectivity of these zoospores in *Solanum lycopersicum*, a host species that has been relatively understudied in relation to *P. cinnamomi* [67–70]. Our results confirm that *Solanum lycopersicum* is a valuable host for future studies involving this phytopathogen.

## Results

### The protocol for the acquiring of *Phytophthora cinnamomi* zoospores stocks

**Fig. 1:**
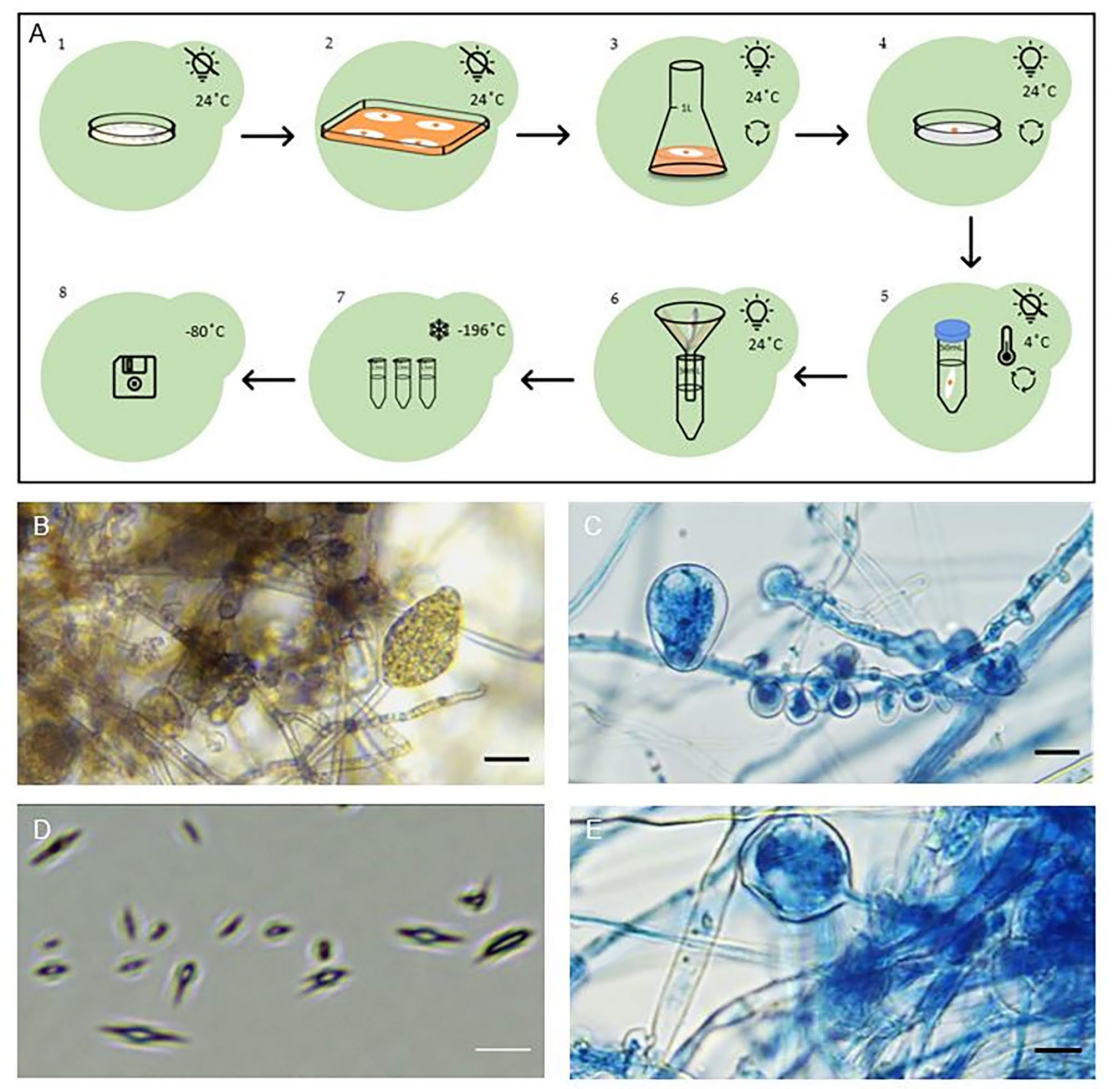
Obtaining *Phytophthora cinnamomi* zoospores. **A.1.***P. cinnamomi* growth in Potato Dextrose Agar medium (PDA) in darkness, at 24 °C for 3 weeks. **A.2.** Inducing mycelium growth by growing *P. cinnamomi* on V8 agar medium in darkness, at 24 °C for 7 days on a miracloth disc. **A.3.** Inducing sporangia formation in V8 clarified liquid medium under fluorescence light and shaking, at 24 °C for 48 h. **A.4.** Second sporangia induction in mineral salt solution, enriched with chelated iron solution, under fluorescent light and shaking at 24 °C for 24 h. **A.5**. Inducing zoospore formation in sterile water under shaking, at 4 °C for 90 min. **A.6.** Filtration of zoospores. **A.7.** Freezing of zoospores in liquid nitrogen. **A.8.** Storage at -80 °C until use. **B.** Bright field of mature sporangium. **C**. *P. cinnamomi* culture stained with trypan blue, correponding to chlamydospore and hyphal swelling after the step of sporangia induction with mineral salt solution. Scale bar = 50 μm. **D**. Bright field snapshot image taken of obtained fresh zoospores. Scale bar = 20 μm. Images were taken at 40x magnification. **E.***P. cinnamomi* culture stained with trypan blue, correponding to chlamydospore and hyphal swelling after the step of sporangia induction with mineral salt solution where some zoospores are observed. Scale bar = 90 μm

Before proceeding with our experimental work, an extensive literature review was conducted to assess the current state of research. Drawing from previous studies, we synthesized a comprehensive global protocol, outlined in Fig. 1 (steps 1 to 8). In this study, we introduce two additional steps to the protocol (Fig. 1), involving the rapid freezing of zoospores in liquid nitrogen followed by storage at -80 °C (Fig. 1, steps 7 and 8). These additions allowed us to procure zoospores for ongoing investigations into *Phytophthora cinnamomi* host interactions.

Fresh zoospores were obtained based on previously published methodologies [6, 50, 52, 53, 55, 65] (detailed in Fig. 1, Sect. 5), and the development of *P. cinnamomi* cultures was monitored with snapshots captured at various stages. Figure 1B illustrates fresh mature sporangia, while Fig. 1C depicts chlamydospore and hyphal swelling following sporangia induction with a mineral salt solution. Additionally, Fig. 1D provides a snapshot of free-living zoospores obtained during the process. The observed results and morphology align with previous studies describing *P. cinnamomi* under similar growth conditions and are consistent with the identification criteria outlined by the European and Mediterranean Plant Protection Organization (OEPP/EPPO, Bulletin 34, 155–157, 2004, PM 7/26) for *P. cinnamomi*.

### Assessment of *Phytophthora Cinnamomi* zoospore viability via absorbance measurement

**Fig. 2:**
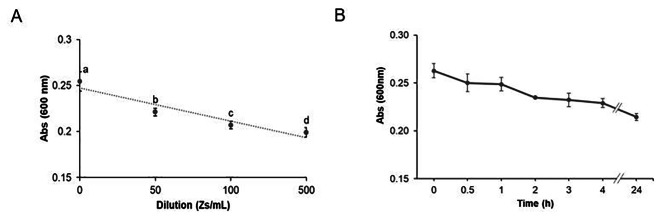
Viability of *Phytophthora cinnamomi* zoospores measured via MTT and Neubauer chamber. (**A**) The scatter plot shows the concentration of zoospores (×10^8^/mL), at 50, 100 and 500 dilutions, counted using a Neubauer chamber plotted against the absorbance signal (measured at 600 nm using MTT; see Sect. 5). Significant differences were based on one-way analysis of variance (ANOVA) with a variance check (*p* < 0.05). Lowercase letters (a, b, c and d) indicate significant differences (*R* = 0.907). Error bars indicate standard deviation (SD) (*n* = 20). (**B**) Changes in zoospore survival at 23 °C measured via MTT. Significant differences were based on a non-parametric Krustal-Wallis´s test with a variance check (*p* < 0.05). Error bars indicate standard deviation (SD) (*n* = 14). The data were analyzed using the Stat-graphics Centurion 19 program

The MTT method, detailed in Sect. 5, enabled the measurement of zoospores at concentrations as low as 10 [6] Zs/ml, aligning closely with results obtained through manual counting using a Neubauer chamber (Fig. 2A). Particularly effective for assessing zoospores survival at room temperature (approximately 23 °C), the MTT method provided stable measurements over 24 h, exhibiting a slight decline in survival without significant statistical variance across measured time points (Fig. 2B). Leveraging an absorbance plate reader, MTT facilitated the simultaneous measurement of multiple samples and triplicates, streamlining assay procedures while minimizing the residual error inherent in manual counting methods. Throughout our study, both the MTT method and manual counting via the Neubauer chamber were employed to ensure comprehensive data collection and analysis.

### Determining viability of fresh *Phytophthora cinnamomi* zoospores using fluorescence analysis

**Fig. 3:**
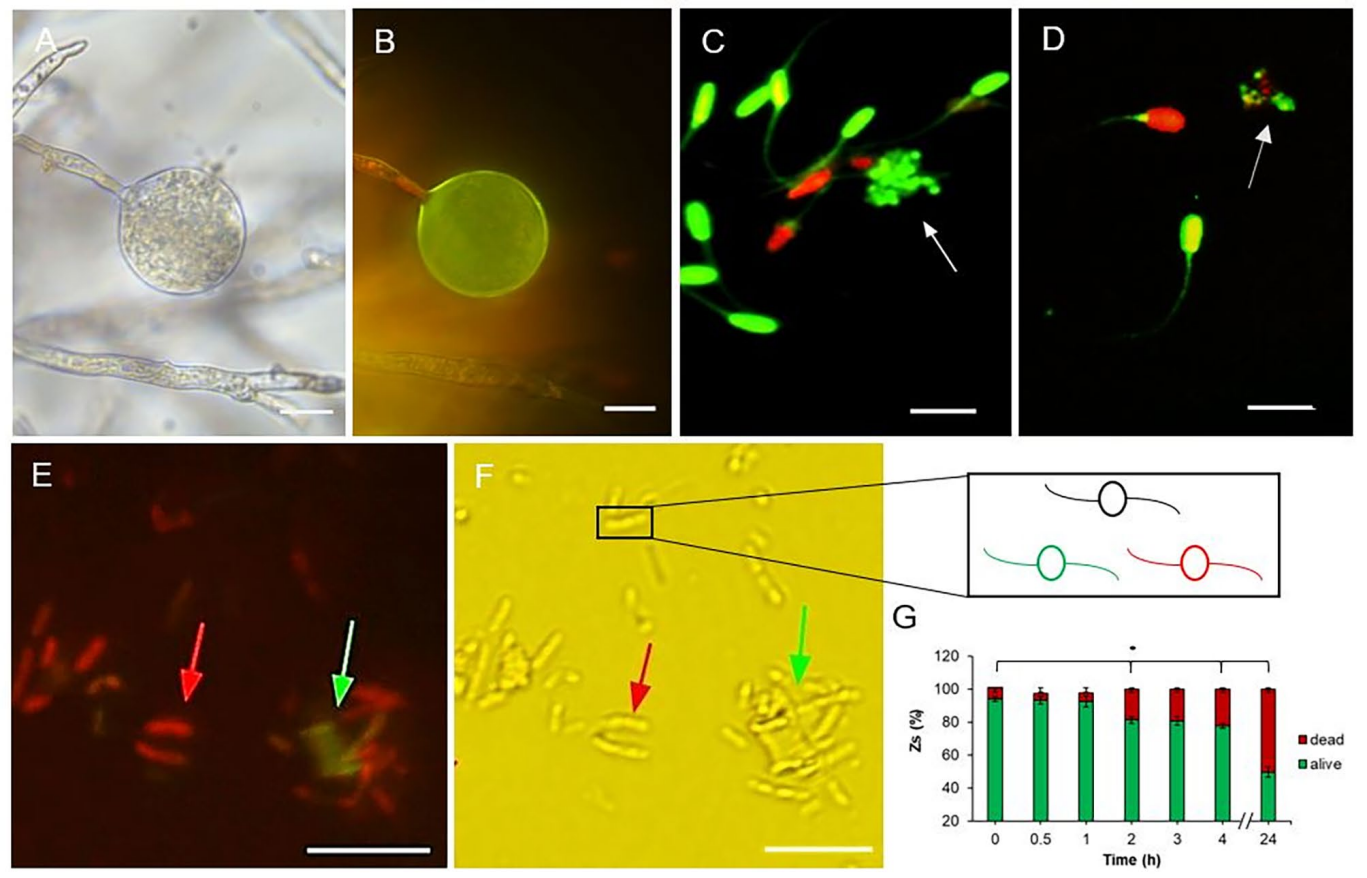
Viability of *Phytophthora cinnamomi* cells determined by fluorescence. (**A**) Bright image of chlamydospore and hypha. (**B**) fluorescent image of A. Scale bars = 50 μm. (**C**) Snapshot of zoospores (white arrowed), and Duroc sperm cells at time 0 by SQS® system. (**D**) Snapshot of zoospores (white arrowed), and Duroc sperm cells after 1 h by SQS® system. Scale bars = 30 μm. **E** and **F**. Bright (left), and fluorescence (right), images of zoospores by SQS® system. **F inset.** Detail that alive cells are arrowed in green while dead cells are arrowed in red. Scale bars = 20 μm. Images with 40x magnification. **G**. Percentage of zoospore survival after 0, 0.5, 1, 2, 3, 4, and 24 h, determined by taking at least five snapshots per time, measured with SQS® machine and counted with ImageJ® Software (see Sect. 5). Error bars indicate standard deviation (SD) (*n* = 35). The data were analyzed with Stat-graphics Centurion 19 program, with a variance check (*p* < 0.05) indicated by one asterisk

The dispersal of Phytophtora cinnamomi zoospores in water and their subsequent adhesion to host roots are intricately linked to their motility [39, 41, 45, 46, 71]. Hence, we monitored the cell viability of chlamydospores, hyphae, and zoospores in culture, utilizing both bright field microscopy (Fig. 3A) and fluorescence imaging (Fig. 3B, detailed in Sect. 5). To assess live and dead cells, porcine spermatozoa were introduced into the medium (refer to Sect. 5), with zoospores indicated by arrows (Fig. 3C and D). Zoospore viability is depicted in Fig. 3E and F, with a corresponding schematic representation aiding identification (inset F). Analysis of fluorescence data obtained via SQS® (Fig. 3G, detailed in Sect. 5) revealed a slight increase in the percentage of decayed cells after 24 h, aligning with observations from MTT assays (Fig. 2B). This increase was attributed to residual mitochondrial activity detected on red cells by SQS®. Notably, a strong correlation was observed between the green fluorescence signal and zoospore viability and motility. Conversely, zoospores exhibiting red fluorescence lacked motility during the assays, suggesting that this method enables the determination of zoospore mobility and survival, like estimates made using porcine spermatozoa. A comparison between the counting methods is provided in Figure S1A.

### Survival and recovery of *Phytophthora cinnamomi* zoospores following freezing conditions

**Fig. 4:**
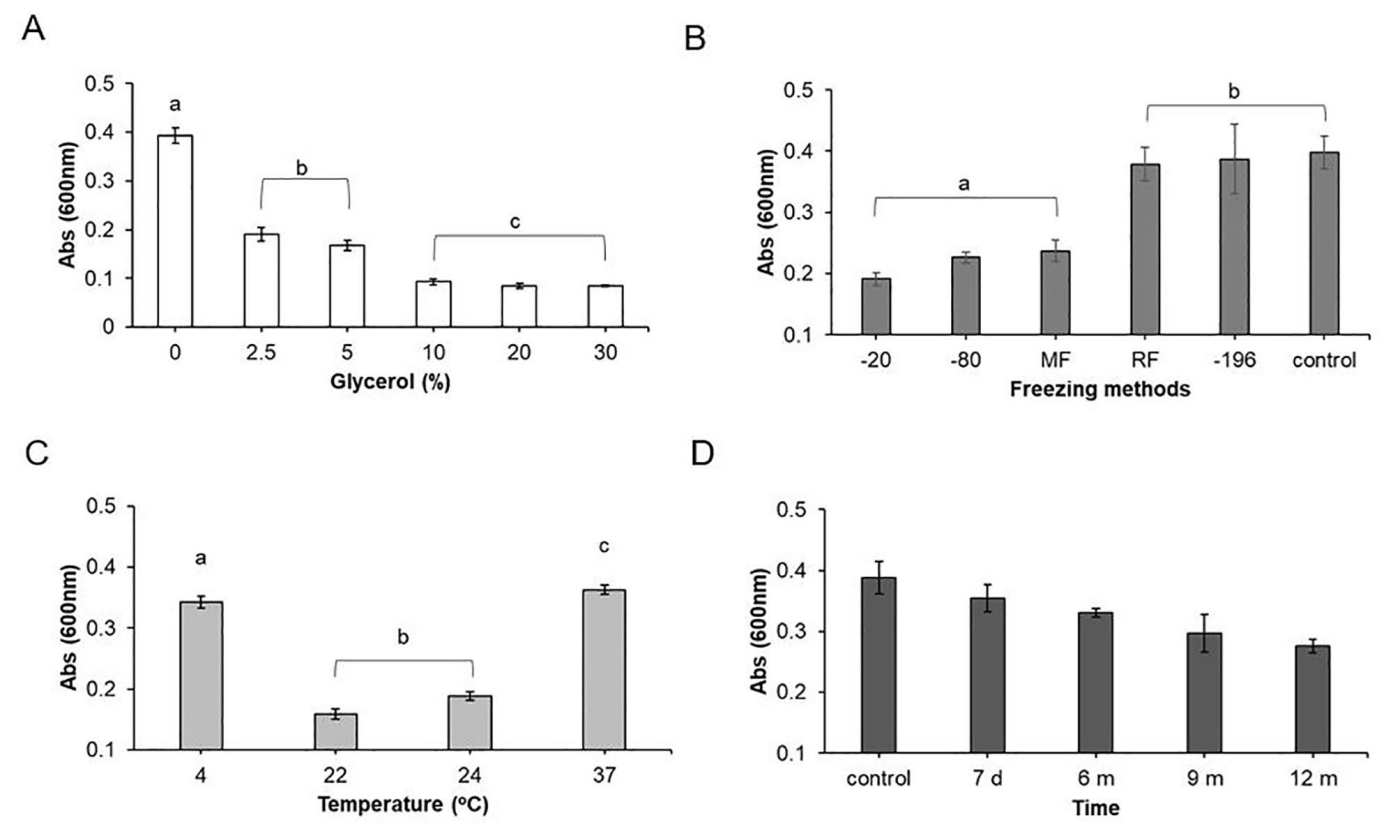
Survival of *Phythophthora cinnamomi* zoospores to freezing and defrosting. (**A**) Survival of zoospores after freezing in glycerol (0, 2.5, 5, 10, 20, and 30%). Lowercase letters (a, b, and c) indicate significant differences. Error bars indicate standard deviation (SD) (*n* = 115). (**B**) Survival of zoospores using next freezing methods: −20 °C (− 20), − 80 °C (− 80), Mister Frosty (MF), ramp freezing (for 22–18 − 4 to − 20 °C) (RF), liquid nitrogen (− 196 °C). For A and B significant differences were based on one-way analysis of variance (ANOVA) with a variance check (*p* < 0.05). Lowercase letters (a and b) indicate significant differences. Error bars indicate standard deviation (SD) (*n* = 16). (**C**) Survival of zoospores after defrosting at 4, 22, 24, and 37 °C. Lowercase letters (a, b, and c) indicate significant differences. Error bars indicate standard deviation (SD) (*n* = 30). (**D**) Survival of zoospores stocks at seven days (d) and six, nine and twelve months (m). The zoospore stock analyzed was 2 × 10^8^ /mL. At least four independent biological assays were performed with similar results. Error bars indicate standard deviation (SD) (*n* = 14). For C and D significant differences were based on Krustal-Wallis´s test with a variance check (*p* < 0.05). All data were analyzed using the Stat-graphics Centurion 19 program

To establish stable stocks of zoospores, we investigated optimal conditions for obtaining non-fresh zoospores with maximal recovery capacity. The survival of non-fresh zoospores exhibited an inverse relationship with glycerol concentrations ranging from 0 to 30%, with the highest survival rate observed in the absence of glycerol (Fig. 4A). Further kinetic studies over 60 min at 4 °C confirmed the decline of zoospores viability in the presence of glycerol, attributed specifically to glycerol and not to freezing or ice treatments, both in non-fresh zoospores (Figure S2A) and fresh zoospores (Figure S2B).

The recovery of zoospore survival post-freezing was assessed using the MTT assay (Fig. 4B). Freezing methods involving liquid nitrogen and sequential freezing (RF) demonstrated the highest survival rates at 80%, while other methods yielded less than 60% survival. Specifically, freezing fresh zoospores in a water solution without glycerol, in liquid nitrogen, emerged as the optimal and most efficient method for conserving zoospore stocks.

Furthermore, among the various defrosting temperatures tested (4 °C, 22 °C, 24 °C, and 37 °C), zoospores exhibited higher survival rates upon defrosting at 4 °C and 37 °C. However, defrosting at 4 °C (on ice) was deemed the simplest and quickest method for laboratory settings, as it requires less time and energy compared to 37 °C, which necessitates a water bath or thermoblock (Fig. 4C).

Long-term survival of zoospore stocks at -80 °C was evaluated over periods ranging from seven days to one year using the MTT assay, revealing a slight decrease in survival of 2.86% (± 0.5) per month (Fig. 4D).

### Retention of viability and infectivity in non-fresh *Phytophthora cinnamomi* zoospore stocks post-freezing

**Fig. 5:**
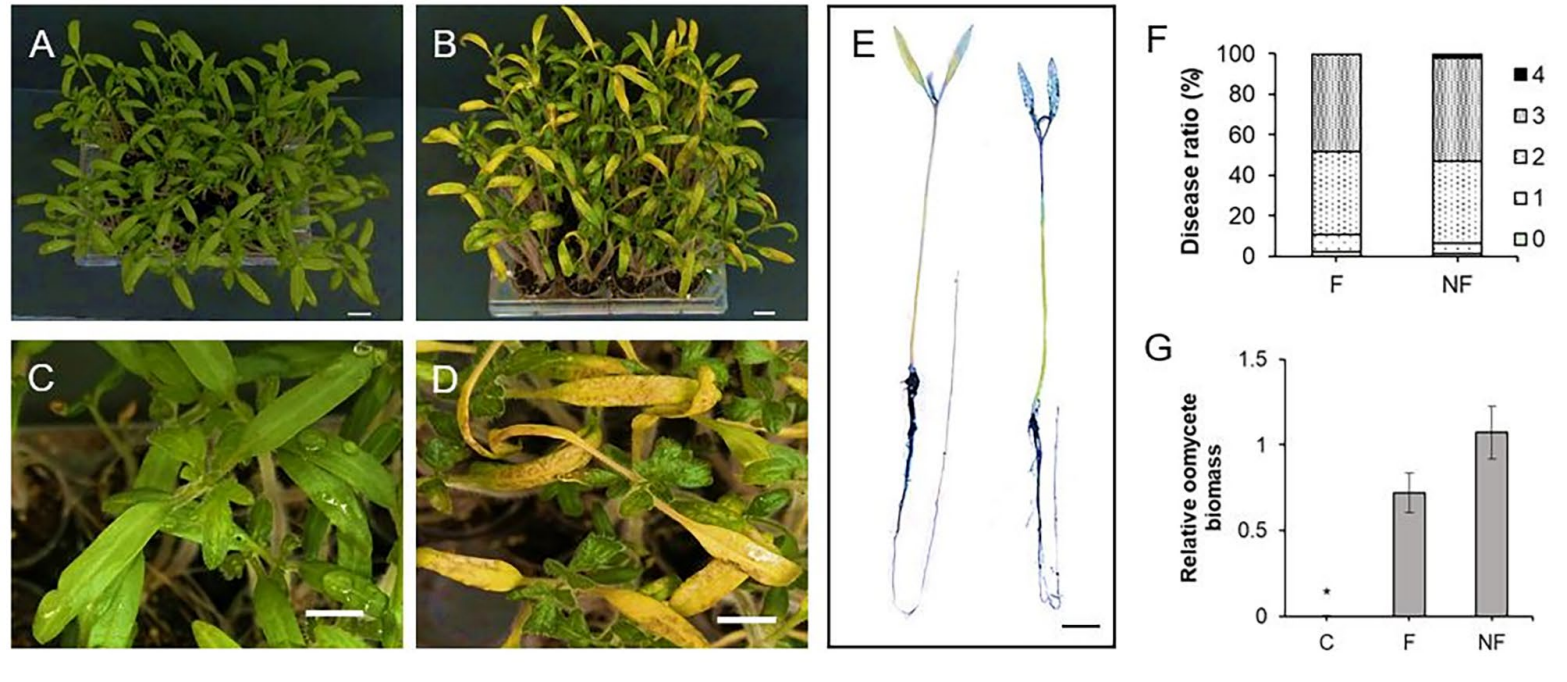
Symptoms of *Solanum lycopersicum* seedlings inoculated with *Phytophthora cinnamomi* zoospore stocks. Fourteen-day-old seedlings were grown on soil and inoculated with a final concentration of fresh zoospores (F), or non-fresh zoospore stocks (2 × 10 ^7^ Zs/ml, see Sect. 5). **A** and **C**. Control plants (water). **B** and **D**. Symptoms of inoculated plants after four days of inoculation (4 dpi). Scale bar = 1 cm. **E.** Necrotic lesions detected by trypan blue staining, control (left image), and inoculated plant (right) at 4dpi. Scale bars = 1 cm. **F.** Disease ratio at 4 dpi, on seedlings performed with F, and NF. Symptom severity was assessed on a scale where “0” is absence of symptoms, “1” plant growth inhibition, “2” light chlorosis on leaves, “3” significant chlorosis on leaves and root necrosis, “4” high necrosis on leaves and roots and decayed seedlings. (*n* = 240). **G.** Oomycete biomass was quantified into vegetal tissue at 4dpi, inoculated with F (fresh) and NF (non-fresh) zoospores, measured by qRT-PCR using specific primers for *P. cinnamomi β-Tubulin* and normalized to the *Solanum lycopersicum β-actin* (see Sect. 5). The data were analyzed using the Stat-graphics Centurion 19 program, with a variance check (*p* < 0.05) indicated by asterisk. Error bars indicate standard deviation (SD) (*n* = 3)

To assess the infectivity of *Phytophthora cinnamomi* zoospore stocks, we developed a rapid inoculation method using *Solanum lycopersicum* seedlings. *Solanum lycopersicum* has been reported as a host for *P. cinnamomi* [4, 67–69, 72], enabling swift evaluation of zoospore infectivity. Fourteen-day-old *Solanum lycopersicum* seedlings were inoculated with previously frozen zoospore stocks directly onto soil. Within just 4 days of inoculation, the zoospore stocks induced high levels of root necrosis and chlorosis on primordial leaves compared to water-treated controls (Fig. 5A and C depict controls, while Fig. 5B and D show inoculated samples). Necrosis in seedlings was further confirmed through trypan blue staining (Fig. 5E).

### Quantification of *Phytophthora cinnamomi* burden in plants and re-isolation using qRT-PCR

We assessed the infectivity of fresh Phytophthora cinnamomi zoospores compared to zoospore stocks by quantifying the Phytophthora cinnamomi burden via qRT-PCR (refer to Sect. 5). Our findings revealed a slight increase in disease severity symptoms in plants inoculated with zoospore stocks (Fig. 5F) compared to those inoculated with fresh zoospores (Figure S3A and C for controls, and Figure S3B and D for inoculated plants). Confirmation of necrosis on seedlings inoculated with both fresh zoospores and zoospore stocks was demonstrated through trypan blue staining (Figure S3E).

The quantification of oomycete biomass was conducted via qRT-PCR on tissues inoculated with fresh *P. cinnamomi* zoospores (F) and zoospore stocks (NF), as well as on non-inoculated control plants (C), with Ct values of *β-tubulin* (*P. cinnamomi*) normalized to *β-actin* (*Solanum lycopersicum*) housekeeping genes (see Sect. 5). The *β-actin* housekeeping gene for *Solanum lycopersicum* was previously published [73], while a β-tubulin housekeeping gene specific to *Phytophthora cinnamomi* was designed for this study (refer to Table S1). Prior to analysis, efficiencies and melting curves were determined to confirm primer specificity for both genes (Figure S4A and S4B for *β-tubulin* gene, and Figure S4C and S4D for *β-actin* gene).

Although slight differences were observed in plant disease symptoms (Fig. 5F), no statistically significant disparities were found in oomycete burden between the two types of inoculations (Fig. 5G).

Additionally, qRT-PCR was conducted to amplify mycelium re-isolated from inoculated *Solanum lycopersicum* seedlings (see Sect. 5). Representative photos of *P. cinnamomi* mycelium growth on plates, obtained from the roots after seven days, are presented in Figure S5A and Figure S5B. A photo of the current stock of *P. cinnamomi* mycelium is shown in Figure S5C. Staining of mycelium recovered from roots with trypan blue is depicted in Figure S5D. These images were compared to the current mycelium and with previous descriptions of *P. cinnamomi* hyphae, confirming the identity of *P. cinnamomi* [74, 75]. The melting curve obtained by qRT-PCR, from amplification performed using *P. cinnamomi* mycelium obtained from plates, or mycelium recovered from *Solanum lycopersicum* roots, were identical, confirming a unique amplicon, and showing that *P. cinnamomi* mycelium recovered from *Solanum lycopersicum* corresponds to *P. cinnamomi* (Figure S5E and Figure S5F). Oomycete biomass quantified in nanograms is presented in Figure S5G.

## Discussion

As part of our study, we conducted a comprehensive biochemical and physiological investigation to identify optimal laboratory conditions for cultivating viable and virulent *Phytophthora cinnamomi* zoospores. Throughout this process, we discovered that each developmental stage of *P. cinnamomi* required specific environmental conditions, often mirroring, or resembling its natural habitat. We identified four key factors that significantly influenced the growth of *P. cinnamomi*: the plant content of the growth medium, light exposure, the physical state of the medium (solid or liquid), and temperature.

In the initial phase of mycelial growth, we found that a high concentration of plant nutrients was crucial for enhancing hyphal development. Interestingly, complete filtration of the growth medium resulted in the inhibition of mycelial growth, indicating that elevated levels of specific plant nutrients are conducive to the survival and proliferation of *P. cinnamomi* on culture plates. Additionally, our investigations revealed that the composition of the soil substrate exerted a profound influence on the survival of *P. cinnamomi*, further emphasizing the importance of environmental factors in its growth and development [6, 76].

V8 juice, a widely consumed commercial juice blend, serves as the current medium for cultivating *P. cinnamomi* and comprises extracts from eight distinct plant tissues. Our observations revealed that *P. cinnamomi* growth on potato dextrose agar (PDA), which solely contains nutrients derived from potatoes, was significantly lower compared to growth in V8 juice. This disparity underscores the necessity for a rich availability of plant-derived nutrients in the laboratory medium for robust *P. cinnamomi* growth. This observation likely aligns with the necrotic activity exhibited by *P. cinnamomi*, facilitated by various enzymes at different developmental stages [12, 13, 39, 43, 45, 46, 71, 77].

A notable illustration is the necrosis instigated by alpha-cinnamomin during the initial phases of vascular colonization [29, 68, 78–80]. Comparative trials involving *P. cinnamomi* inoculations with both zoospores and mycelium in *Quercus ilex* revealed a correlation between elevated infection levels of *P. cinnamomi* and reduced lignin content alongside increased nutrient levels derived from necrotic tissue. This correlation suggests that nutrient availability may play a role in the subsequent formation of chlamydospores [13]. Additionally, another study demonstrated that the proliferation of *P. cinnamomi* was associated with the presence of detritivores in nutrient-rich soil environments [81, 82]. Zoospores exhibited an extended survival period by colonizing deceased plant tissue, although outcomes varied depending on the host [83]. Furthermore, it would be intriguing to explore how plant nutrients contribute to *P. cinnamomi* virulence in the artificial medium utilized in laboratory settings.

The second crucial factor influencing the growth of *Phytophthora cinnamomi* was the physical state of the medium, whether solid or liquid. Initial steps for mycelium growth were conducted on solid surfaces, with the mycelium cultured on filter paper discs, akin to the solid surfaces of roots where mycelium naturally proliferates. Conversely, attempts to cultivate mycelium in liquid media hindered its development. Conversely, the production of zoospores necessitated liquid media, inhibiting zoospore production on solid media (data not shown). These conditions mirrored the natural growth environment of zoospores in water.

The third factor of significance was light exposure. Specifically, during the sporangia induction stage, the importance of light for sporangia induction varied among previous studies, with some highlighting its significance while others did not [55, 84, 85]. In this study, the initial stages of *P. cinnamomi* growth were conducted in darkness, aligning with the natural conditions of *P. cinnamomi* growth below ground. It was noted that the presence of low light levels in forested areas contributes to the development of *P. cinnamomi* [86]. Under our experimental conditions, we found that the presence of light was crucial, particularly for the induction of sporangia. These observations were substantiated through trypan blue staining during the process. Specifically, the presence of light during the transfer of the paper disc with mycelium from the V8 solid plate to a liquid medium was necessary for sporangia induction. Additionally, continuous shaking proved to be essential. Conversely, the absence of light or shaking notably reduced sporangia production and subsequent zoospore production. Some studies [87–89] have determined that alternating light and dark periods during sporangium production increase the growth of *P. cinnamomi*. Additionally, several authors have noted that *P. cinnamomi* can persist as a saprophyte on dead plant tissue [83]. Our observations suggest that the optimal conditions for *P. cinnamomi* development overlap with those found in artificial greenhouses [88], contributing to the occurrence of *P. cinnamomi*. Furthermore, the abundance of *P. cinnamomi* in Mediterranean forest soils has been linked to a predictable spatial structure associated with heavily defoliated oak trees and light exposure in forests [49]. However, direct comparisons with laboratory conditions may not be meaningful. Given the contrasting conclusions regarding the influence of light on *P. cinnamomi* sporangiogenesis, further investigation into the role of light is warranted. It’s worth noting that sporangia development occurs belowground in the absence of light under optimal conditions in nature [85].

The final critical factor in *Phytophthora cinnamomi* growth to obtain zoospores was temperature. The induction of zoospores development from sporangia was conducted as a later step, adapting methods previously published [44, 54, 55, 90, 91]. The highest efficiency for obtaining fresh zoospores from sporangia cultures was achieved by maintaining them in darkness within a cold-water bath (refer to methods Sect. 5). In contrast, assays conducted at room temperature or under light conditions yielded significantly lower numbers of fresh zoospores. These cold-water conditions mirror those found in nature, particularly during spring and autumn, facilitating the development of zoospores within sporangia before their release into above- or below-ground water sources.

After establishing the optimal conditions for growing *Phytophthora cinnamomi* and obtaining fresh zoospores, we investigated the survival of zoospores under various freezing and thawing conditions. While the MTT method has been traditionally utilized for quantifying protozoa [92], it has not been applied to quantify *P. cinnamomi* zoospores previously ^6^. In our study, we found this method to be highly useful, particularly when dealing with numerous samples and replicates, as it enables the measurement of zoospores in a 96-well plate reader.

Furthermore, we assessed the motility of zoospores using SQS®, employing two fluorescent dyes in conjunction with sperm cells. This method proved effective in determining the size of zoospores and their survival under different conditions. Notably, we observed that zoospores remained viable for up to 24 h at room temperature (23 °C), a significant extension compared to the 4-hour survival period reported in previous studies [93, 94]. This prolonged survival time may be attributed to the different conditions utilized to obtain zoospores in our study.

Previous research investigating the persistence of *Phytophthora cinnamomi* DNA and RNA in various soil types revealed that *P. cinnamomi* RNA, a real-time indicator of the metabolic activity of living *P. cinnamomi*, remained detectable up to one day after soil inoculation, whereas DNA persisted at later stages of detection [8]. Although direct comparison between these two studies is challenging due to the presence of other *P. cinnamomi* spores besides zoospores in soil, it is worth considering that the survival of zoospores in laboratory settings and in nature may be longer than initially assumed.

One of the most surprising findings of this study was the remarkable survival of non-fresh *P. cinnamomi* zoospores under freezing conditions, even in the absence of cryoprotectants, particularly glycerol. In nature, *P. cinnamomi* is highly sensitive to extreme temperatures, but not warm temperatures [5, 6, 9, 30, 31, 91, 95, 96]. The unexpected survival of zoospores in liquid nitrogen, without the use of antifreeze agents, was attributed to the toxicity of the glycerol medium, especially to fresh zoospores, compared to non-fresh ones (refer to Figure S2). This result was surprising in light of previous studies on *P. cinnamomi* [55, 97–100].

Conventionally, cryopreservation of Phytophthora species cultures involves immersion in a cryoprotectant solution such as dimethyl sulfoxide (DMSO), 1,2-propanediol, glycerol, or skim milk/glycerol to prevent ice crystal formation during freezing in liquid nitrogen [101, 102]. In fact, the major collections saved for this specie use that method [99, 100]. However, our findings suggest that intracellular ice formation in zoospores may not occur, or its effects on zoospore recovery after freezing may be minimal. Control of the freezing rate is critical to optimize the ice nucleation process and minimize cell damage.

Because rapid freezing leads to intracellular ice nucleation [103, 104], which is lethal to cells, we performed various tests to optimize the freezing method of fresh zoospores. Our experiments to optimize the freezing method of fresh zoospores revealed that slower freezing rates resulted in higher cell death rates, possibly due to prolonged exposure to hypertonic solutions and subsequent “pickling” effects. Similar observations have been reported with green macroalgae solutions of *Enteromorpha intestinalis* [105].

Further investigations will be necessary to confirm our hypothesis regarding intracellular ice formation in zoospores and to analyze the cell wall composition of zoospores to understand the effects of liquid nitrogen, which allows survival without cryoprotectants, as observed in our study.

*Phytophthora cinnamomi* has been documented to elicit changes in the mineral content of infected plant tissues, with notable differences between susceptible and tolerant hosts [106–110]. For instance, in Eucalyptus, a decrease in metal content was observed in susceptible hosts compared to tolerant ones, implying that *P. cinnamomi* absorbs these minerals from the plants [107]. It is conceivable that the same capacity observed in *P. cinnamomi* for uptake of plant nutrients, salts, and minerals from the host may enable zoospores to absorb salts and minerals from water. This process could potentially protect zoospores from ice crystal formation during freezing, thereby explaining their high survival capacity in the absence of cryoprotectants. However, this hypothesis requires experimental validation to confirm its validity.

The zoospore stocks obtained in this study retained their infectivity towards the plant host even after undergoing freezing and thawing processes. Further investigations are ongoing to comprehensively characterize the response of *Solanum lycopersicum* to *P. cinnamomi* at both physiological and molecular levels. Although *Solanum lycopersicum* is not typically considered a host of *P. cinnamomi*, this study demonstrates that exploring this plant-oomycete interaction could serve as a valuable tool for understanding this complex phytopathogen and elucidating the plant’s response to it.

## Conclusions

To our knowledge, this study represents the first published research on obtaining stocks of *Phytophthora cinnamomi* zoospores by freezing in liquid nitrogen and storing them at -80 °C without the use of cryoprotectants. Remarkably, frozen stocks of zoospores maintained their viability to infect plants comparable to fresh stocks. The findings of our research offer valuable insights for researchers aiming to conduct assays with reduced time and cost, while also circumventing challenges associated with the loss of *P. cinnamomi* virulence during growth on plates. The utilization of MTT and SQS® staining methods enables the monitoring of zoospore survival, revealing a higher survival capacity than initially anticipated. Moreover, we have developed a protocol to investigate the interaction between *P. cinnamomi* and *Solanum lycopersicum*, an underexplored plant-pathogen interaction. This protocol holds promise for advancing our understanding of this complex relationship.

## Materials and methods

### Biological material

The *Solanum lycopersicum* seeds (var. *marmande*) were provided by Ramiro Arnedo S.A, La Rioja, Spain. The seeds were stored and maintained at 4 °C until use. *Phytophtora cinnamomi* Rands, was kindly supplied by TRAGSA (Maceda nursery, Orense, Spain), and isolated by the Center for Research and Technology of Extremadura (CYCYTEX), located in Mérida, Spain.

### Detailed protocol for obtaining Phytophthora cinnamomi zoospores *stock*

The protocol was obtained from the specified bibliography [5, 53, 55, 62, 111], with little modifications. The oomycete was obtained from provider and was previously identified as *Phytophthora cinnamomi*, on potato dextrose agar medium plate cultures (PDA, Sigma-Aldrich, St. Louis MO, USA). PDA plates were obtained using 39 g/L and autoclaved at 120° C for 20 min. *P. cinnamomi* on PDA plates was incubated at 24 °C in darkness for one week within a Memmert incubator (INB 500, Madrid, Spain).

For obtaining *P. cinnamomi* subcultures, V8 agar medium plates were prepared as follows. A piece of 1 cm × 1 cm of mycelium was transferred onto a previously 1-mL-watered Miracloth disk (Miracloth EMD Millipore Corp, Darmstadt, Germany) using a plate with a freshly prepared medium with 100 mL/L of fresh V8 juice (Campbell Soup Company, Camden, NJ, USA), supplemented with 15 g/L of Difco agar (Le Pont de Claix, France), plus 2 g/L of calcium carbonate (CaCO_3_, Sigma-Aldrich, USA), and 0.02 g of β -sitosterol dissolved in 5 mL of ethanol (Sigma-Aldrich, St. Louis, MO, USA). The pH was adjusted with sodium hydroxide (NaOH) or potassium hydroxide (KOH, Merck, Darmstadt, Germany) to 6.5, and 0.1 M HEPES (Sigma-Aldrich, St. Louis, MO, USA) [62, 63] to ensure pH stability. Plates were closed with Bemis^Tm^ Parafilm^Tm^ (St. Louis, Missouri, USA) and protected from light with aluminium foil. Plates were incubated for 12–30 days [62], and growth into an Aralab^®^ digital chamber (Lisbon, Portugal). Controlled conditions included 50% humidity (*v/v*), a temperature of 24 °C during the day and 18 °C during the night, with a 16 h light/8 h dark photoperiod and light intensity of 150 μE·m^− 2^ per second; the photoperiods during the day were fixed and mimicked a spring day based on a meteorology statal agency (AEMET) profile, Madrid, Spain. Those conditions fit with natural conditions used for growing plants (see below).

The diameter of mycelium as well as the high of it, was measured been between 0.5 and 0.9 cm of high and 7–9 cm of diameter accordingly with previous works [112]. To induce sporangia, the Miracloth disc was aseptically transferred to a 1-L Erlenmeyer flask, containing 100 mL of a 10% clarified V8 liquid medium. The medium was then filtered with one slide filter paper, and the filtrate was diluted 10 times with distilled water and autoclaved, following previous work [113]. The Erlenmeyer containing the Miracloth disc, was incubated in an orbital shaker (100 rpm) at 24 °C, under fluorescence light, for 48 h to induce sporangial production. These conditions were selected specifically, after the revision of different protocols [45, 55, 62, 113, 114]. The obtention of sporangia was confirmed via trypan blue (TB) staining and visualization under a light microscope (see TB details below). During this step we observed that the use of smaller Erlenmeyer flask (0.5 L) reduced the oxygen availability for the *P. cinnamomi*, thus decreasing survival. The centrifugation of the clarified medium also diminished the final yield of the process. The same step in the absence of light rendered no or significantly less available alive sporangia.

A second step of sporangia induction was subsequently performed based on previous work [55], the clarified V8 liquid medium was decanted off, and the Miracloth disc with the *P. cinnamomi* mycelium obtained was transferred with sterilized forceps onto a 9-cm sterilized plastic Petri plate (Fisher Scientific SL, Madrid, Spain). The disc was then washed by carefully adding 20 mL of a previously sterilized and fresh mineral salt solution (MSS). The MSS medium was prepared into an Erlenmeyer with 0.01 M of calcium nitrate (CaN_2_O_6_), 0.005 M of potassium nitrate (KNO_3_) and 0.004 M of magnesium sulphate heptahydrate (MgSO_4_ 7H_2_O), all chemicals were obtained from Sigma-Aldrich, St. Louis, MO, USA. The MSS was autoclaved and then supplemented with 1 mL/L of a chelated iron solution (CIS). To prepare CIS, 13.05 g/L of Ethylene-dinitrile-acetic acid (EDTA, Sigma-Aldrich, St. Louis, MO, USA) was mixed with 7.5 g/L of potassium hydroxide (KOH, Merck, Darmstadt, Germany), 24.9 g/L of iron sulphate (II), and heptahydrate (FeSO_4_ 7H_2_O, 99%, both from Sigma-Aldrich, St. Louis, MO, USA. The CIS solution was filtered using a 0.22-μm diameter Millipore filter (Sigma-Aldrich, USA) before adding to MSS. 3MM (Micropore^™^, Neuss, Germany), and Bemis^Tm^ Parafilm^Tm^ (St. Louis, Missouri, USA) were used to avoid losing the liquid medium during orbital shaking at 100 rpm for 24 h at 24 °C with fluorescence light. The obtention of MSS and CIS was performed following [55]. MSS and CIS mediums were found essential for sporangia induction. Again, variations of this step, including conducting the process in the absence of light, or transferring the disc to 1 L Erlenmeyer using 100 mL of the medium, rendered, much lower numbers of final zoospores (data not shown).

For the induction of zoospores, the disc containing the *P. cinnamomi* sporangia obtained was transferred carefully with forceps to a 50-mL Falcon tube (Corning Inc, Corning, NY, USA) containing 20 mL of sterile water type I (available for molecular biology, water supllier Aristerra 2011 S.L., Pamplona, Spain), at 4 °C. The tube was then placed in an orbital shaker and kept at 100 rpm at 4 °C for 1.5 h and in the absence of light. The resulting solution (step six at Fig. 1A) was filtered through three slides of a sterilized gauze pad (Hartmann S.A., Heidenheim, Germany); this step was based specifically on [111].

The occurrence of zoospore releasing from sporangia was confirmed using the light microscope and via TB staining (see TB details below). The number of zoospores per ml after release occurs, was measured for each assay, at Neubauer chamber (Marienfeld GmbH & Co. KG, Lauda-Königshofen, Germany), the quantification of zoospores rendered always between 1.8 and 5 × 10^8^ zoospores/ml. A more restrictive filtering with Whatman filter paper for a 110-mm size pore, (GE Healthcare life science, Amersham, UK) rendered much lower quantity of zoospores (data not shown). The number of sporangia per unit of area (mm^2^), on each assay under the microscope, was measured been between 5 and 10 sporangia/mm^2^.

After previouslly described procedures for zoospore acquisition, the long-term maintenance and conservation of *P. cinnamomi* Rands mycelium, was made by taking 1 × 1 cm squares of mycelium from plates where zoospores were obtained. These squares are carefully placed into 1.5 ml Safe-lock Eppendorf tubes (Eppendorf A.G, Hamburg, Germany), rapidly frozen in liquid nitrogen, and subsequently stored at -80 °C. To initiate mycelium reactivation, the tubes are gently thawed on ice and the mycelium is transferred onto fresh Petri dishes containing V8 agar. The cultures are then maintained in darkness at 24 °C for 7 days before start a new process for zoospore obtention.

### MTT procedure to measure the viability of *Phytophthora cinnamomi* zoospores

Because fresh zoospores move extremely fast, currently, for counting, the zoospores need to be fixed to avoid their mobility. This process currently involves killing the cells, or induction of cysts, by centrifugation or vortex [6, 39, 46, 75]. At this point we observed that zoospores turned to cyst after shaking and remained alive, however, we still observed that not all of them turned to cyst and several remains alive moving at Neubauer chamber during counting.

Because that, and to ensuring a good correlation between the counting of alive cells and the final number of zoospores available for inoculation assays, we used one additional method for counting zoospores. A colorimetric assay, based on the absorbance signal produced by formazan, during the mitochondrial oxidative activity produced in the presence of MTT (3-(4,5-dimethylthiazol-2-yl)-2,5-diphenyltetrazolium bromide, Sigma-Aldrich, Rockford, IL, USA) production [92, 115]. This reaction is produced only in viable cells [116].

This method is commonly used to also measure the cell viability in various species of protozoa, including the human pathogen *Leishmania sp*. parasite [117] and has been used to measure viability of *Phytophthora* oospores [6]. Briefly, 100 μL of MTT at 0.5 mg/mL were added to 900 μL solution of zoospores, incubating the mixture at 37 °C for 2 h. Next, 100 μL of 10% Sodium dodecyl sulphate (SDS, Sigma-Aldrich, Rockford, IL, USA) in distilled water was added and the mixture was kept at 37 °C for 2 h. The absorbance of the solution was measured at 600 nm using a spectrophotometer (Hach DR 2000, Hach Co. Loveland, CO, USA). Controls were performed in the same manner but using water instead of zoospores. The straight pattern showing the survival of spores at different dilutions measured via MTT is shown at Figure S1A.

### SQS^®^ fluorescence procedure to detect the viability of *Phytophthora cinnamomi* zoospores

Cell viability of zoospores, chlamydospore and hyphal structures was conducted using the Swine Sperm Viability (SQS^®^) & Morphology Kit (Arquimea, S.L., Madrid, Spain). This methodology is currently used for determining the survival and detect the motility of different sperm cells [118]. The kit employs a dual staining approach that enables for the differentiation of live and dead sperm cells and flagella, as well as organ abnormalities [119]. To determine correlation between motility and viability of fresh zoospores, they were stained using this fluorescence dye, as controls for the alive and dead cells, pig spermatozoids were added at the medium with zoospores. This method is based on the green or red fluorescence signal emission produced by live or dead cells, respectively, in real-time.

Briefly, a mixture of 1 μL of Duroc sperm cells, used for reference of size and survival, and 2 μL of a zoospore solution (2 × 10^8^ Zs/mL) was prepared and added simultaneously to the SQS® plate. Two distinct methods were utilized for analyzing and detecting the fluorescence signal. The first method involved an SQS2 semen analysis system machine (kindly provided by ARQUIMEA S.L., Madrid, Spain), coupled with a fluorescence microscopy-based sperm counter, integrated with a high-resolution CMOS camera with a high-power blue LED computer vision system. This system was used to evaluate the total number of cells taking snapshots at different times, using at least five photos per time. A SQS2 red led signal corresponded to Em617 nm/Ex536 nm, and a green led signal to Em526 nm/Ex500 nm. A second detection was performed by using a fluorescence stereomicroscope A292/21 Microscopy iScope IS.3153-PLFi/6 with Fluorescence–IS.3153-PLi/6,nEWF 10×/22; Plan Fluarex PLFi, 4×, 10×, 20×, 40×, 60×; 100× oil lenses that included blue, green, UV-DAPI, and red fluorescence filters; and a charge-coupled device (CCD) digital cooled camera A292/21 Euromex 20 MP USB 3.0 with a 1-inch CMOS sensor (Microsercon SLU, Madrid, Spain). The green led signal corresponded to Em570 nm/Ex510–550 nm, and the red led signal to Em690 nm/Ex620 nm. At least 10 photographs were captured per condition. Image processing and quantification of trypan blue were performed using the ImageJ® program and plug-in tools [120, 121] as previously [120] (Figure S1 B to D).

### Frosting and thawing of fresh *Phytophthora cinnamomi* zoospores

The most frequent method for conserving microbial stocks for long periods of time within laboratories is glycerol imbibition prior to a fast freezing into liquid nitrogen afterwards [101, 122–125]. Cryopreservation methods were tested based on previous studies made with liquid nitrogen [102, 126–132] and they also included the addition of glycerol as a cryoprotectant [133, 134].

To study the direct effect of the glycerol on the zoospore survival, two kinds of assays were performed with zoospore stocks, non-fresh zoospores (NF) and fresh zoospores (F). Aliquots of 250 μL of zoospore stocks were defrosted in ice (4 °C), and mixed with 250μL of glycerol solutions in water, at final percentages of 0, 5, 25, and 50%. The survival of zoospores was measured at 0, 30, and 60 min using MTT. In a second kind of assay, 250 μL aliquots of F zoospores were treated as previously and were immediately placed into liquid nitrogen, before defrosting in ice. The recovery capacity was measured at times 0, 30 and 60 min, by MTT.

To determine the best method for frosting zoospores, they were immersed in water, in the absence of glycerol, and frozen under different conditions. The conditions used were based on a previous work performed on *Phytophthora hibernalis, P. infestans, P. lateralis and P. nicotianae* [133]. A direct immersion into liquid nitrogen (− 196 °C). A sequence ramp treatment at − 20 °C and at − 80 °C. A one degree decreasing ramp of freezing using the Nalgene^®^ Mr Frosty™ Cryocontainer (C1562, Sigma-Aldrich, Rockford, IL, USA), used to achieve freezing with a speed of 1 °C/min following the manufacturer’s instructions, briefly, the container was filled with isopropyl alcohol before the samples were placed in a vial holder, with the assembly then being moved to a − 80 °C freezer overnight. In addition, a ramp freezing was used for getting sequential water baths, performed over periods of two hours at 23 °C, 18 °C, 4 °C, with a final freezing for 18 h at − 20 °C with a final step at − 80 °C (RF). For determining the survival of zoospores after different conditions of freezing, zoospores were slowly thawed on ice, as well as thawed in water baths at 22, 24, and 37 °C for 30 min. The recovery and survival rates for the zoospore stocks after freezing were measured for twelve months. In all methods, 1.5 mL Safe-lock Eppendorf tubes (Eppendorf A.G, Hamburg, Germany) were used. The tubes were then stored in a freezer at − 80 °C until evaluation of the viability of them.

### Plant growth and plant inoculation conditions

*Solanum lycopersicum* seedlings were grown following the methods we optimized previously [135] with little modifications. Briefly, the seeds were germinated in soil in twelve-well microplates of twelve pots (Deltalab S.L, Barcelona, Spain), into a sterile mixture of Projar Professional Seed Pro 5050, NPK 14–16 − 18 plus microelements (Commercial Projar S.A., Valencia, Spain) substrate and vermiculite (3:1), with a granulometry measurement of between 0 and 3 mm (Commercial Projar S.A., Valencia, Spain), with 10 seeds per pot and twelve pots per plate, with a total of 120 seedlings per plate. In all assays, at least 120 *Solanum lycopersicum* seedlings were used for each assay and condition. The plants were irrigated with a regular water supply and grown for 18 days in an Aralab^®^ digital chamber (Lisbon, Portugal). Controlled conditions included 50% humidity (*v/v*), a temperature of 24 °C during the day and 18 °C during the night, with a 16 h light/8 h dark photoperiod and light intensity of 150 μE·m^− 2^ per second for all experiments; the photoperiods during the day were fixed and mimicked a spring day based on a State Meteorological Agency (AEMET) profile, Madrid, Spain.

For plant inoculations, after fourteen days, seedlings were inoculated with a 500-μL of a sterilized distilled water suspension of fresh or non-fresh zoospores (2 × 10^7^ Zs/mL), detailed on each assay. Control samples were treated with 500 μL of sterilized distilled water. The inoculated seedlings were located into a digital growth chamber at same conditions for growth (Aralab S.L, Lisbon, Portugal) following disease symptoms.

### Plant disease symptoms

Disease symptoms of *Solanum lycopersicum* seedlings were followed from 0 to 12 days after inoculation or until plant decayed, currently apparent disease symptoms on roots and secondary symptoms produced on leaves, were observed after three or four days. The analysis of the seed germination rate and disease symptoms on *Solanum lycopersicum* seeds and seedlings was made according to the Protocol for tests on distinctness, uniformity and stability stablished by Community Plant Variety Office (CPVO-TP/1-Rev-5, 01/06/2021). The plant disease symptoms produced by *P. cinnamomi* were considered following the standards approved for diagnostic protocols for regulated pests by EPPO council (European and Mediterranean Plant Protection Organization), at PM 7/26, OEPP/EPPO Bulletin 34, 155–157, 2004). Those symptoms included principally root rot brown lesions and necrosis produced directly by the pathogen and secondary symptoms of decline producing leave chlorosis and later necrosis and dead. The disease ratio was determined as the percent of disease severity symptoms present on the seedlings related to the total number of plants under the same treatment, this scale is detailed as follows: “0” no disease symptoms; “1” delayed growth observed for shoots and roots; “2” chlorosis on the aerial part; “3” significant chlorosis and necrosis both on leaves and roots; “4” high necrosis and decayed seedlings. For each scale, the percentages of disease severity symptoms were calculated related to the total number of plants (n), considering “n” the 100%. Delayed growth related to controls, when was observed by view on aerial parts or roots, was confirmed digitally by analysing snapshots from harvested seedlings, using Image J® [120, 121]. The cell death was detected by trypan blue (TB) staining, as previously described [136]. Briefly, a TB solution was prepared with 10 mL of lactic acid (85%), 10 mL of phenol (TE balanced buffer, pH 7.5–8.0), 10 mL glycerol (≥ 99%), 10 mL of distilled water, and 40 mg of TB (final concentration of 10 mg/mL). The plant tissue or *P. cinnamomi* culture samples stayed for 20 min in the solution before been rinsed with 100% ethanol overnight and preserved in 60% glycerol at 4 °C or mounted on slides until observation under bright-light microscopy using a stereomicroscope (Microsercon SLU, Madrid, Spain), to obtain digital photos and snapshots. Image processing of snapshots was performed, when necessary, using the ImageJ® program using the specific corresponding software and plug-in tools [120, 121] as previously [120].

### *Phytophthora cinnamomi* burden and re-isolation in plants measured by qRT-PCR

In addition, at this work we were focus and interested in the quantification of pathogen biomass, in planta, useful to determine the oomycete burden into the tissue, because this is a crucial step when monitoring disease resistance [137–140].

With the unique objective of identifying and quantifying the oomycete *P. cinnamomi* burden on infected plants using qRT-PCR, we selected a specific gene previously utilized for *P. cinnamomi* identification. This gene, initially identified by Weerakoon et al. in 1998 [141] for studying *Tubulin* expression and microtubule organization in *P. cinnamomi*, was cloned, and sequenced from a cDNA library. The gene, found in genomic DNA without introns, encodes a 445-amino acid protein with a single copy in the genome. Comparison of this *P. cinnamomi β-Tubulin* gene sequence with *β-Tubulin* genes from various fungi, algae, and protists supported the hypothesis that Oomycetes are more closely related to algae and protists than higher fungi.

Subsequently, Kroon et al. in 2004 [142] used this gene in a phylogenetic analysis of Phytophthora species based on mitochondrial and nuclear DNA sequences to identify *P. cinnamomi.* They designed two oligonucleotides based on the previously published genomic DNA sequence by Weerakoon et al. in 1998 and amplified a 989 DNA bases pair fragment, demonstrating its utility for *P. cinnamomi* identification. Bezuidenhout et al. in 2010 [141] also employed this gene for taxonomic identification and phylogenetic marker of *P. cinnamomi*, studying Phytophthora taxa based on previous gene studies.

Briefly, for relative quantification by qRT-PCR, of oomycete burden on plants, the tissues of *Solanum lycopersicum* seedlings, growth on soil, inoculated or not, were harvested with eighteen days, corresponding to four days after inoculation with zoospores (4 dpi), or treated with water on controls. The tissues were weighted (g), snap frozen and finely ground to powder with a Sylamat® (Ivoclar Vivadent, Liechtenstein, Austria). Total RNA extraction, including DNAase treatment, was isolated from 0.350 g of plant tissue, using at least two plants per triplicate, and at least three triplicates per treatment, following manufacture instructions (NZY Total RNA Isolation kit, Nyztech, Lisbon, Portugal). The quantity and quality of RNAs were obtained using a nanodrop (UV-Vis ACTG Gene UVS − 99. 200 an 850 nm), and a Qubit 4.0 (Fisher Scientific, Madrid, Spain) respectively. RNA samples were visualized in 1% agarose gel stained with Gel Red (Nippon, Japan). First-strand cDNA synthesis was performed using an hexanucleotide random primer, following manufacture instructions of First-Strand Synthesis Kit (Amersham-Pharmacia, Rainham, UK).

The method used for relative quantification of oomycete burden on inoculated and non-inoculated seedlings was determined by qRT-PCR based on previous works [143–145]. Briefly, 1μL cDNA obtained (as before), from inoculated and non-inoculated plants, were analysed by qRT-PCR using specific housekeeping oligonucleotides of plant and mycelia. The relation between Ct numbers obtained measuring β-*actin* (*Solanum lycopersicum*) and *β-tubulin* (*P. cinnamomi*) amplification was calculated. Data were acquired using the One-Step PCR Applied Biosystem Analysis software (Version 2.01), and changes in transcript levels were determined using the 2^−∆∆CT^ method [146].

The nuclear *β-Tubulin* gene of *P. cinnamomi* corresponds to Accession No. U22050, GenBank accession number GU1911391 (selected from *P. cinnamomi* genome database (PHYCI_418545, https://fungidb.org/fungidb/app/), gene-specific primers were designed considering the annealing temperatures of 56 °C (forward) and 58 °C (reverse), that amplified one product of 160 bp with an efficiency of the 97,4%. These specific primers resulted useful for amplification and identification of the *P. cinnamomi β-Tubulin* gene using qRT-PCR for the first time (Table S1), resulting in a single product profile in the qRT-PCR melting curve. The gene was only amplified in cDNA from mycelia or inoculated *Solanum lycopersicum* seedlings and was absent in control non-inoculated plants, confirming its specificity to *P. cinnamomi* and allowing us to quantify the burden on plants using a previously published housekeeping gene used on *Solanum lycopersicum*. The *β-Actin* gene-specific primers for *Solanum lycopersicum* corresponds to (*Solyc11g005330.2*, https://solgenomics.net/) and were obtained from previous work [73] with annealing temperatures of 60 °C (forward) and 57 °C (reverse), amplifying one product of 150 bp with an efficiency of the 98,01%(Table S1).

The qRT-PCR was performed using a SYBR® Green qPCR master mix (Nzytech, Lisbon, Portugal), using a final volume of 20μL per well. The reaction mixture was 1μL aliquot of the first-strand cDNA, 10μL of SYBR® Green mix, 1μL of each oligonucleotide (10μM) and 7μL of DEPC-Water (Ambion^™^, Austin, USA). The qRT-PCR program was adjusted to melting curve oligonucleotides temperatures and consisted of 10 min at 25 °C, 30 min at 50 °C, and 5 min at 85 °C. Samples were run into a DNA Engine One-Step qRT-PCR machine (Thermofisher Scientific, USA).

The primers were verified for specificity using a Basic Local Alignment Search Tool (BLAST at NCBI). The program of qRT-PCR consisted of 3 min at 95 °C, 40 cycles of 30 s at 95 °C, and 30 s at 60 °C, with a final extension step consisting of 7 min at 72 °C with a final dissociation melting curve. Data points were compared using t-tests. At least, three independent biological replicates obtained from different assays, were used with three technical replicates in each experiment.

Simultaneously, to confirm *Phytophtora cinnamomi* re-isolation from *Solanum lycopersicum*, assays of absolute qRT-PCR were performed. Briefly, roots from inoculated seedlings were harvest after seven days as previously. The roots were surface sterilized with water and transferred onto a Petri dish containing PDA medium. Subsequently, the dishes were placed into a digital chamber Aralab® (Lisbon, Portugal), under the current conditions described before. Plates were kept in darkness for seven days allowing mycelium growth. One 1cmx 1 cm piece of agar containing the mycelium was transferred onto a PDA and used to re-isolate mycelium, sporangia and zoospores (following Fig. 1A). In parallel, a fresh mycelium stock of *P. cinnamomi* was obtained as before. In both cases, 0.135 g of *P. cinnamomi* mycelium were snap frozen in liquid Nitrogen and homogenized to powder with a Sylamat® (Ivoclar Vivadent, Liechtenstein, Austria). The extraction of total RNA and First-strand cDNA were performed as previously. The absolute quantification of mycelia was made by qRT-PCR relating *β-tubulin* Ct to ng of mycelia cDNA.

### Statistical analysis

The Stat Graphics Centurion XVI.II program (Stat Point Technologies, Inc., Warrenton, VA, USA) was used for all data analysis using a Variance check (*p* value > 0.05). Statistical significance was calculated for all the experiments by unpaired t-test (*P* < 0.05), indicated with one asterisk (*), bars with non-asterisk are not significantly different, according to one-way ANOVA, Bonferroni’s multiple comparison test (*P* > 0.05). For qRT-PCR analysis, error bars indicate standard deviation (SD) (*n* = 3). Bars with the same letter are not significantly different according to one way analysis of variance (ANOVA), Bonferroni’s multiple comparison test (*P* < 0.05). Linear trend estimation was calculated by t-test (*P* < 0.05), when the correlation coefficient is significantly different the linear trend is shown with gray dots, correlation degree (“r), is shown at the corresponding figure legend.

### Electronic supplementary material

Below is the link to the electronic supplementary material.

Supplementary Material 1

Supplementary Material 2

## Data Availability

Availability of data and materials: All materials generated in this study are available from the corresponding author upon request.
