# Supplementary material for: An improved method to study Phytophthora cinnamomi Rands zoospores interactions with host
Source: BMC Plant Biol. 2024 Jun 6;24:508. doi: 10.1186/s12870-024-05205-2 (PMC11154991; doi:10.1186/s12870-024-05205-2)

**Figure Legends**

**Figure S1. Counting methods of *Phytophthora cinnamomi* zoospores.** **A.** Survival of the Zs obtained with Neubauer chamber, SQS® fluorescence (SQS), and MTT. **B.** Snapshot corresponding to live and dead Zs obtained by SQS®. **C.** Counting the Zs using ImageJ® of C. **D.** Snapshot of Zs at Neubauer chamber. The data were analyzed using the Stat-graphics Centurion 19 program, significant differences based on Krustal-Wallis´s test with a variance check (p < 0.05). Lowercase letters (a, b, c, and d) indicate significant differences. Error bars indicate standard deviation (SD) (n = 21). Data were obtained from at least three independent biological assays (see Section 5). White scale bars = 50 µm. Black scale bar = 1 mm. Images correspond to 40x magnification.


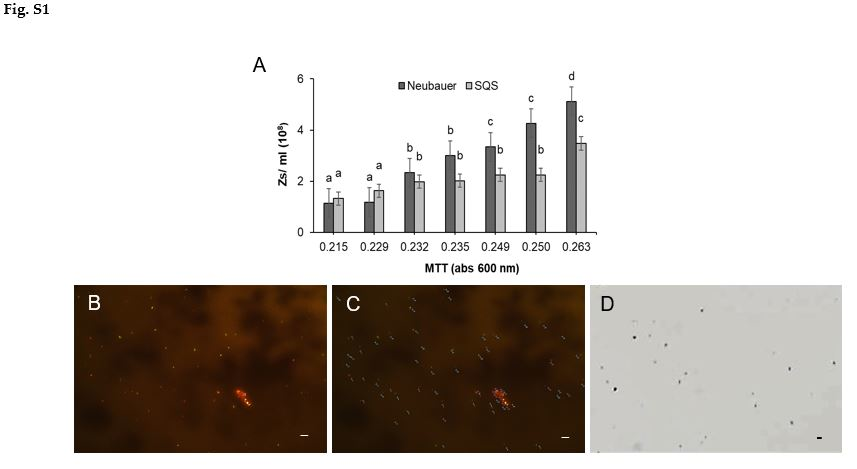


**Figure S2. Response of *Phytophthora cinnamomi* zoospores to glycerol.** **A.** Survival of NF Zs at 0, 30, and 60 minutes (min), measured by MTT. Lines represent the presence of glycerol at 0 (blue), 5 (red), 25 (gray) and 50% (yellow). The kinetic process involved the thawing of the Zs on ice with subsequently addition of glycerol. **B.** Survival of fresh Zs at 0, 30, 60 minutes, and 4 hours, measured using MTT (see methods) at 600 nm. The data presented are derived from four independent biological assays, all assays were performed with Zs at 4 °C. The data were analyzed using the Stat-graphics Centurion 19 program, significant differences based on Krustal-Wallis´s test with a variance check (p < 0.05) indicated by one asterisk. Error bars indicate standard deviation (SD) (n = 36).


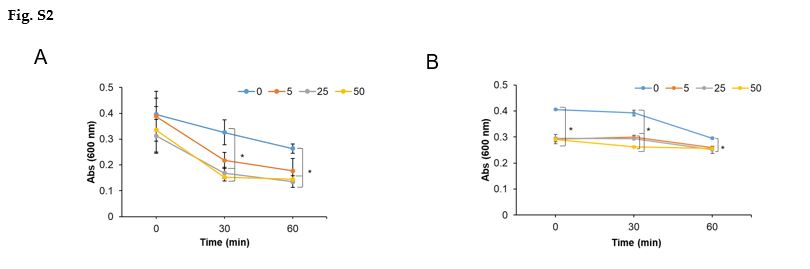


Figure S3. Symptoms of *Solanum lycopersicum* seedlings inoculated with fresh *Phythophthora cinnamomi* zoospores. A-D. Four-teen-day-old seedlings grown in soil: A, C: controls; B, D: seedlings inoculated with 2x10^7^ Zs/ml after 4 days (dpi). Scale bars = 1 cm. E. *Solanum lycopersicum* seedlings stained with Trypan blue after 4 dpi, control on the left and infected on the right. Scale bar = 1 cm.


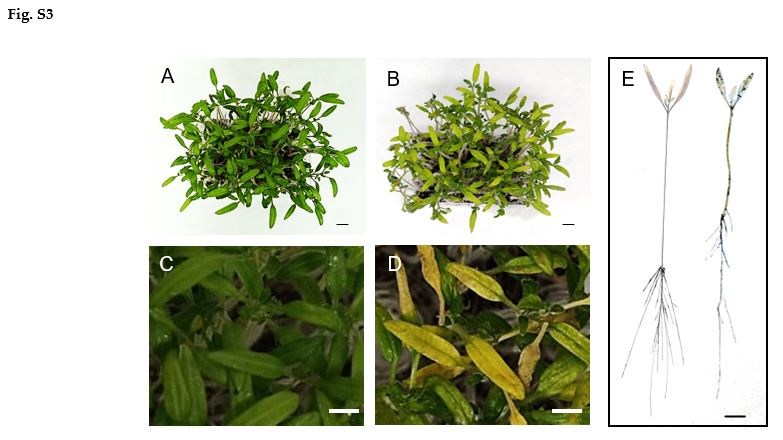


Figure S4. Quality control of oligonucleotides used to quantification and re-isolation of *Phytophthora cinnamomi*. A-B. Efficiency and dissociation curve corresponding to *β-Tubulin* hpuskeeping gene of Pc. C-D. Efficiency and dissociation curves corresponding to *Actin* gene of *Solanum lycopersicum*. Amplification data were obtained by qRT-PCR using tissue from fourteen-day-old seedlings, after four days of inoculation (2x 10^7^ Zs/ml), (see Section 5).


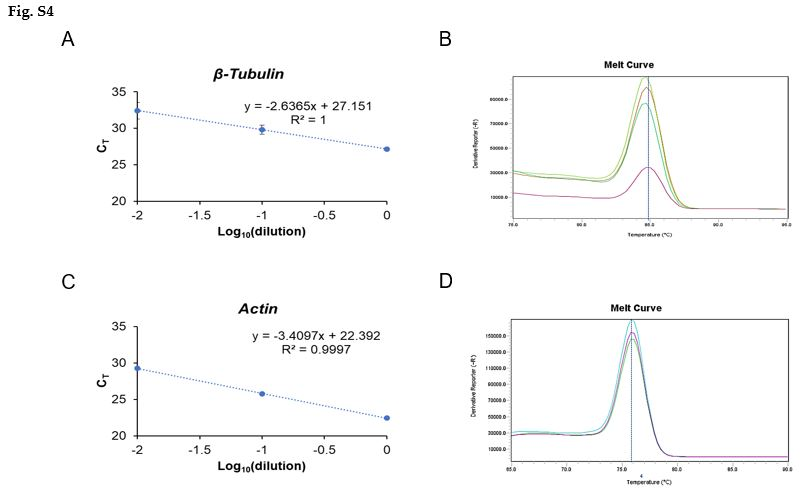


**Figure S5. Isolation and re-isolation of *Phytophthora cinnamomi* mycelium from *Solanum lycopersicum* infected roots.** Roots were harvested from *Solanum lycopersicum* seedlings previously inoculated with 2x10^7^ Zs/ml after 7 dpi. Root tissue was water surface sterilized before location on PDA medium. **A.** Mycelium growth from roots after 3 days. **B.** Mycelium growth from roots after 7 days. Scale bars = 1 cm. **C.** Mycelium of Pc obtained following the current protocol described at 5.3 section after 7 days. Scale bar = 1 cm. **D.** Trypan blue staining of Pc mycelium (black arrowed), obtained on B. Scale bar = 100 µm. **E.** Dissociation melting curves corresponding to *β-tubulin* gene amplified from mycelium recovered from *Solanum lycopersicum* roots. **F.** Dissociation melting curves corresponding to *β-tubulin* gene amplified from mycelium obtained on PDA. **G**. Quantification of oomycete biomass (ng x 10^3^), re-isolated from *Solanum lycopersicum* infected roots (R) and isolated from mycelium PDA plate (M), (see Section 5). The data were analyzed using the Stat-graphics Centurion 19 program, with a variance check (p < 0.05) indicated by asterisk. Error bars indicate standard deviation (SD) (n = 3).


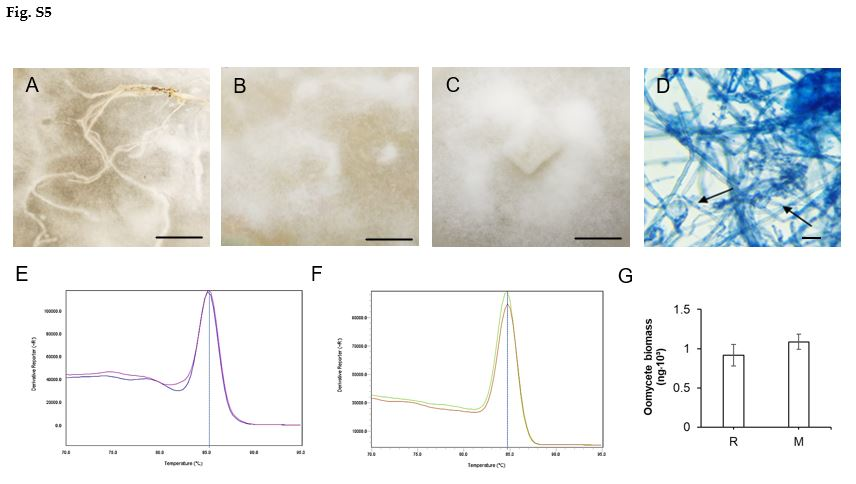

Supplement: Supplementary file 1 — Supplementary Material 1 [file 12870_2024_5205_MOESM1_ESM.docx]
