# Supplementary material for: An improved method to study Phytophthora cinnamomi Rands zoospores interactions with host
Source: BMC Plant Biol. 2024 Jun 6;24:508. doi: 10.1186/s12870-024-05205-2 (PMC11154991; doi:10.1186/s12870-024-05205-2)
Supplement: Supplementary file 2 — Supplementary Material 2 [file 12870_2024_5205_MOESM2_ESM.pdf]

Table S1. qRT-PCR primers used in this study.

Gene symbol	Polarity	Primer sequence (5´-3´)	Efficiency	Amplicon lenght	Reference
β-Tubulin	forward	CGCTGTCCGTGCACCAGCTTG	97,4 %	160 bp	this work
β-Tubulin	reverse	CATGTCCGGCATCACCACGTGC	97,4 %	160 bp	this work
β-Actin	forward	CAAGTTATTACCATTGGTGCTGAGA	98,01 %	150 bp	Martín-Trillo *et al.* 2011
β-Actin	reverse	TGCAGCTTCCATACCAATCATG	98,01 %	150 bp	Martín-Trillo *et al.* 2011
